# RGD peptide and graphene oxide co-functionalized PLGA nanofiber scaffolds for vascular tissue engineering

**DOI:** 10.1093/rb/rbx001

**Published:** 2017-02-07

**Authors:** Yong Cheol Shin, Jeonghyo Kim, Sung Eun Kim, Su-Jin Song, Suck Won Hong, Jin-Woo Oh, Jaebeom Lee, Jong-Chul Park, Suong-Hyu Hyon, Dong-Wook Han

**Affiliations:** 1 Department of Cogno-Mechatronics Engineering; 2 Department of Optics and Mechatronics Engineering; 3 Department of Nanoenergy Engineering, College of Nanoscience and Nanotechnology, Pusan National University, Busan 46241, Korea; 4 Cellbiocontrol Laboratory, Department of Medical Engineering, Yonsei University College of Medicine, Seoul 03722, Korea; 5 Center for Fiber and Textile Science, Kyoto Institute of Technology, Matsugasaki, Kyoto 606-8585, Japan

**Keywords:** RGD peptide, graphene oxide, poly(lactic-co-glycolic acid), biofunctional scaffold, vascular smooth muscle cell

## Abstract

In recent years, much research has been suggested and examined for the development of tissue engineering scaffolds to promote cellular behaviors. In our study, RGD peptide and graphene oxide (GO) co-functionalized poly(lactide-*co*-glycolide, PLGA) (RGD-GO-PLGA) nanofiber mats were fabricated via electrospinning, and their physicochemical and thermal properties were characterized to explore their potential as biofunctional scaffolds for vascular tissue engineering. Scanning electron microscopy images revealed that the RGD-GO-PLGA nanofiber mats were readily fabricated and composed of random-oriented electrospun nanofibers with average diameter of 558 nm. The successful co-functionalization of RGD peptide and GO into the PLGA nanofibers was confirmed by Fourier-transform infrared spectroscopic analysis. Moreover, the surface hydrophilicity of the nanofiber mats was markedly increased by co-functionalizing with RGD peptide and GO. It was found that the mats were thermally stable under the cell culture condition. Furthermore, the initial attachment and proliferation of primarily cultured vascular smooth muscle cells (VSMCs) on the RGD-GO-PLGA nanofiber mats were evaluated. It was revealed that the RGD-GO-PLGA nanofiber mats can effectively promote the growth of VSMCs. In conclusion, our findings suggest that the RGD-GO-PLGA nanofiber mats can be promising candidates for tissue engineering scaffolds effective for the regeneration of vascular smooth muscle.

## Introduction

In recent years, much research has focused on the development of novel combinative approaches to support cellular behaviors. As part of an effort to promote cellular behaviors, tissue engineering has emerged and many studies have been suggested and examined for the fabrication of tissue engineering scaffolds [1–5]. An ideal scaffold should not only provide an appropriate microenvironment for the growth of cells, but should also guide the cellular behaviors. Therefore, there have been intense efforts to design artificial scaffolds having the capability to promote cellular behaviors. Electrospinning is a simple and promising technique for creating biomimetic scaffolds because the electrospun nanofiber mats is quite similar to the natural extracellular matrix (ECM) in structural aspects [6, 7]. Moreover, the properties of electrospun nanofiber mats can be easily controlled by adjusting electrospinning parameters, such as voltage, polymer composition and flow rate [1, 8, 9]. Consequently, electrospun fiber mats have been widely employed as artificial scaffolds [10, 11].

Poly(lactide-*co*-glycolide, PLGA) is a fairly well-known and usually utilized biodegradable polymer for manufacturing tissue engineering scaffolds due to its outstanding biocompatibility, biodegradability, appropriate physicochemical and thermal characteristics, and long clinical experience [12, 13]. Therefore, PLGA-based nanofiber mats have been extensively explored in the context of tissue engineering scaffolds [14–16]. Herein, we co-functionalized RGD peptide and graphene oxide (GO) into the PLGA nanofiber mats. The RGD peptide is a tripeptide sequence (Arg-Gly-Asp) and a major recognition motif for integrin. Therefore, RGD peptide has received significant attention as a functional ligand for enhancing cellular behaviors. It has been reported that the RGD peptide can enhance cell adhesion, which leads to promote cell growth [17]. However, although RGD peptide is a promising bioactive material, it is limited to using RGD peptide owing to its complicated synthesis process [18–20]. To address this limitation, we used M13 phage as an organic building block to functionalize RGD peptides into the PLGA nanofibers. The M13 phage is a widely-used filamentous virus that can express numerous desired proteins on its side wall surface. The 2700 major coat proteins (pVIII) compactly cover the side wall of M13 phage and can be genetically modified to express desired proteins, which leads to massive production of RGD peptides [3, 5, 21–23]. In addition, M13 phage can be simply and effectively amplified by infecting bacteria, followed by the mass amplification technique [21, 22]. Hence, RGD peptide functionalization by utilizing RGD peptide-expressing M13 phages (RGD-M13 phages) is very efficient. On the other hand, GO, an oxidized graphene, has superior thermomechanical properties [24]. Moreover, it has been revealed that the GO can not only promote cell growth, but can also enhance thermomechanical properties of polymer substrates [24–27]. Therefore, GO has attracted particular attention as a novel nanofiller in polymer substrates [4, 28, 29].

In the present study, the RGD peptide and GO co-functionalized PLGA (RGD-GO-PLGA) mats were prepared via electrospinning from a mixed solution of PLGA, GO and RGD-M13 phage. The RGD-GO-PLGA mats were analyzed by scanning electron microscopy (SEM), contact angle measurements, Fourier-transform infrared (FTIR) spectrometry, thermogravimetric analysis (TGA), and *in vitro* degradation analysis. Furthermore, the growth behaviors of primarily cultured vascular smooth muscle cells (VSMCs) on the RGD-GO-PLGA nanofiber mats were evaluated in order to explore their potentials as artificial tissue engineering scaffolds for the regeneration of vascular smooth muscle.

## Materials and methods

### Preparation of RGD-GO-PLGA nanofiber mats

The functionalization of RGD peptides into the electrospun fiber mats was accomplished by utilizing RGD-M13 phages as reported in our previous studies [3, 30]. The RGD peptides were expressed on the side wall of M13 phage by genetic engineering. Briefly, an inverse polymerase chain reaction cloning method was conducted to express RGD peptides on major coat proteins of M13 phages, as describe elsewhere [5, 23, 30].

RGD-GO-PLGA mats were produced by electrospinning technique. The electrospinning solution was prepared by dissolving PLGA (lactide/glycolide molar ratio = 75/25, molecular weight = 70 000–110 000 Da, 200 mg/ml, Evonik Industries, Essen, Germany) and RGD-M13 phages (10 mg/ml) in 1, 1, 1, 3, 3, 3-hexafluoro-2-propanol (Sigma-Aldrich Co., St Louis, MO, USA), and then GO solution (2 mg/ml, Sigma-Aldrich Co.) in water was blended with RGD-M13 phage and PLGA blend solution. Electrospinning was conducted by loading the RGD-M13 phage, GO and PLGA blend solution in a syringe attached to a 21-gauge needle. A voltage of 14 kV was applied and the blend solution was injected at a feeding rate of 0.2 ml/h. A steel rotating wheel covered by aluminum foil was placed 11 cm from the needle tip to collect the nanofibers. After then, RGD-GO-PLGA mats were kept in vacuum for at least 8 h at room temperature in order to eliminate all remaining solvent.

### Characterization of RGD-GO-PLGA nanofiber mats

Surface morphologies of RGD-GO-PLGA mats were characterized by SEM (S-800, Hitach, Tokyo, Japan) with a 5 kV acceleration voltage. Water contact angles of the mats were measured to investigate the surface hydrophilicity of the mats using a OCA10 goniometer (Dataphysics, Filderstadt, Germany) by placing a drop of distilled water (10 μl) on the electrospun mats. The composition of electrospun mats was analyzed by FTIR spectrometry. The FTIR spectra of each mat were obtained by a Nicolet 560 FTIR spectroscope (Nicolet Co., Madison, WI, USA) with 4.0 cm^−^^1^ resolution and 16 scans ranging from 4000 to 500 cm^−^^1^. To examine the thermal stability of the mats, TGA was performed using a TGA n-1000 analyzer (Scinco Co., Seoul, Korea). The nanofiber mats were approximately weighed 5 mg each in open aluminum pans and heated in the temperature range of 25–500°C at 10°C/min heating rate. The *in vitro* degradation behaviors of mats were analyzed by immersing the PLGA, GO-PLGA, RGD-PLGA and RGD-GO-PLGA mats in Dulbecco’s phosphate-buffered saline (DPBS, Gibco, Rockville, MD, USA) at 37°C. The cumulative weight loss was measured after 1, 2, 3, 4 and 8 weeks.

### Cell culture condition and *in vitro* assays for VSMC behaviors on RGD-GO-PLGA nanofiber mats

VSMCs were isolated from the tunica media of the inferior vena cava of Sprague-Dawley rats and primarily cultured as described elsewhere [31, 32]. The primarily cultured VSMCs were routinely incubated in Dulbecco’s modified Eagle’s Medium (Welgene, Daegu, Korea) containing 10% of fetal bovine serum (Welgene) and 1% of antibiotic antimycotic solution (10 000 U of penicillin, 25 μg of amphotericin B and 10 mg of streptomycin per ml, Sigma-Aldrich Co.) at 37°C and 5% CO_2_ in a humid incubator. Studies were performed with the cells between the third to the seventh passage.

To examine the initial attachment and proliferation of VSMCs on RGD-GO-PLGA mats, a cell counting kit-8 assay (CCK-8 assay, Dojindo, Kumamoto, Japan) was performed following the manufacturer’s protocol. The number of viable cells was directly proportional to the metabolic reaction products obtained in the CCK-8 assay [33, 34]. Briefly, a concentration of 1 × 10^4^ cells/ml was seeded on each mat, and incubated for 4 h (cell attachment) or 1, 3, 5 and 7 days (proliferation) at 37°C. After then, the cells were incubated with CCK-8 solution for another 2 h in the dark at 37°C. The absorbance values were determined by using SpectraMax 340 plate reader (Molecular Devices, Sunnyvale, CA, USA) at 450 nm. The absorbance values of the cells cultured on tissue culture plastics (TCPs) were used as positive controls.

### Immunofluorescence imaging of VSMCs on RGD-GO-PLGA nanofiber mats

For the morphological observations, VSMCs were cultured on PLGA, GO-PLGA, RGD-PLGA and RGD-GO-PLGA mats for 3 days. The cells were then fixed in formalin solution (3.7% of formaldehyde in DPBS, Sigma-Aldrich Co.) for 10 min, and were immersed in 0.1% of Triton X-100 in DPBS (Sigma-Aldrich Co.) for 5 min. A 2% of bovine serum albumin (BSA, GenDEPOT, Barker, TX, USA) was added to block non-specific binding of antibodies for another 30 min, and the cells were incubated with tetramethyl rhodamine isothiocyanate (TRITC)-conjugated phalloidin (at 1:40 in 1% of BSA solution in DPBS; Molecular Probes, Eugene, OR, USA) at room temperature for 20 min. The RGD-M13 phages functionalized in the RGD-PLGA and RGD-GO-PLGA mats were immunofluorescence stained using an α-M13 phage antibody (at 1:250 in 2% of BSA solution in DPBS; Sigma-Aldrich Co.) and a secondary fluorescein isothiocyanate (FITC)-labeled goat α-rabbit IgG (at 1:500 in 2% of BSA solution in DPBS; Abcam Inc., Cambridge, MA, USA) for 2 and 1 h, respectively. The nucleus was stained using 4’,6-diamidino-2-phenylindole (DAPI, 1 μM, Sigma-Aldrich Co.) solution in DPBS. All imaging was carried out using a custom-made two-photon laser fluorescence microscopy and immunofluorescence images analyzed with ImageJ 1.48v software (NIH, Bethesda, MD, USA) [35, 36].

### Statistical analysis

All variables were tested in the three independent experiments, each performed in duplicate on different cultures (*n* = 6). Data are presented as the average ± standard deviation (SD). Prior to statistical analysis, data were analyzed for equality of variances by Levene’s test. Statistical multiple comparisons were performed with the Bonferroni test after preliminary one-way analysis of variance. In all cases, *P* < 0.05 was deemed statistically significant.

## Results and discussion

### Physicochemical and thermal properties of RGD-GO-PLGA nanofiber mats

**Figure 1:**
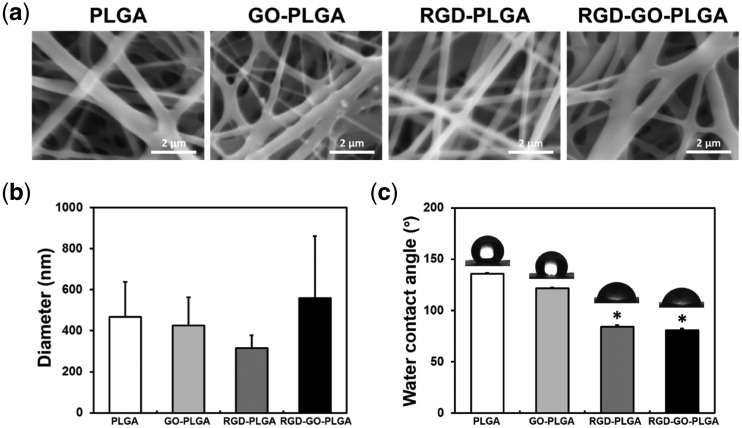
**(a)** Representative SEM images of PLGA, GO-PLGA, RGD-PLGA and RGD-GO-PLGA nanofiber mats. All images shown in this figure are representative of six independent experiments with similar results. **(b)** Average diameters and **(c)** water contact angles of PLGA, GO-PLGA, RGD-PLGA and RGD-GO-PLGA nanofiber mats. An asterisk (*) denotes a significant difference compared with the PLGA and GO-PLGA nanofiber mats (*P* < 0.05). The data are presented as the average ± SD of at least three independent experiments, each performed in duplicate on different samples

The surface morphologies of the RGD-GO-PLGA nanofiber mats were shown to be a 3D network structure resembling the natural ECM (Fig. 1a). The average diameters were 468 ± 170 nm for PLGA mats, 427 ± 137 nm for GO-PLGA mats, 315 ± 62 nm for RGD-PLGA mats, and 558 ± 303 nm for RGD-GO-PLGA mats (Fig. 1b). Regarding the surface area-to-volume ratio, these nanometer-scale fibers allow achieving effective interaction between the RGD-GO-PLGA nanofiber mats and the cells [4]. Therefore, the RGD-GO-PLGA mats can interact effectively with cells because of their superior surface area. Furthermore, the natural ECM is composed of various reticular fibers having dimeters ranging from tens to hundreds of nanometers [37–39]. As shown in Figure 1b, the diameter of the RGD-GO-PLGA nanofibers widely varied in the range from 220 to 860 nm because the RGD-M13 phage and GO were randomly co-functionalized into the PLGA nanofibers. Therefore, the RGD-GO-PLGA mats composed of constituent fibers, which had various fiber diameters, were quite similar to the natural ECM in structural aspects. Figure 1c showed the contact angles of nanofiber mats. The surface hydrophilicity is one of the main factors that contributes to the interaction between cells and substrates. The contact angles were 135.8 ± 0.9° for PLGA mats, 121.5 ± 1.0° for GO-PLGA mats, 83.9 ± 1.7° for RGD-PLGA mats, and 80.6 ± 1.7° for RGD-GO-PLGA mats. Contact angles of the nanofiber mats gradually decreased when GO was functionalized in the nanofibers. This could be attributed to the abundant hydrophilic groups on the GO surface, such as hydroxyl, carboxyl and carbonyl groups [25]. In addition, the contact angles further decreased when RGD-M13 phage was functionalized in the nanofibers. Therefore, the RGD-GO-PLGA nanofiber mats have the most hydrophilic surface among other mats. This increase in surface hydrophilicity of mats can also substantially facilitate the interactions between cells and mats. Therefore, the RGD-GO-PLGA nanofiber mats have a suitable 3D network structure and surface hydrophilic properties that sufficiently fulfill the requirements for tissue engineering scaffolds.

**Figure 2:**
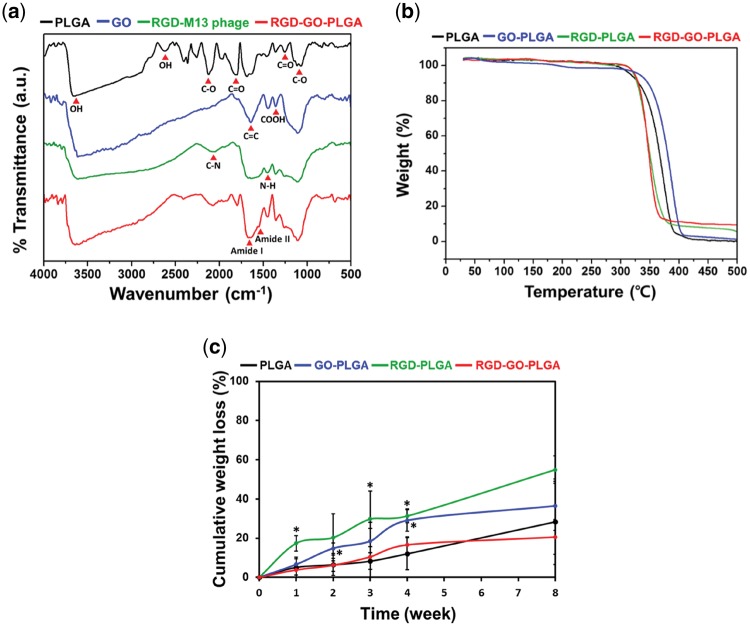
**(a)** FTIR spectra of PLGA, GO, RGD-M13 phage and RGD-GO-PLGA nanofiber mats. **(b)** TGA curves and **(c)** cumulative weight loss of PLGA, GO-PLGA, RGD-PLGA and RGD-GO-PLGA nanofiber mats. An asterisk (*) denotes a significant difference compared with the PLGA nanofiber mats (*P* < 0.05). The data are presented as the average ± SD of at least three independent experiments, each performed in duplicate on different samples

Figure 2a showed the FTIR spectra of PLGA, GO, RGD-M13 phage and RGD-GO-PLGA nanofiber mats. In the spectrum of the RGD-GO-PLGA nanofiber mats, the characteristic peaks of PLGA, GO and RGD-M13 phage were clearly observed. The typical peaks of PLGA were found near 1080 and 1800 cm^−^^1^, which were assigned to the C-O and C = O stretching vibrations of the ester groups, respectively [40]. The specific peaks for GO were also observed at 1110, 1370 and 1640 cm^−^^1^, attributed to the C-O vibrational mode of epoxy group, carboxyl group and C = C bonds of sp^2^ carbon, respectively [41, 42]. On the other hand, the characteristic peaks found around 1550, 1630 and 2080 cm^−^^1^ were corresponded to the amide II, I and C-N vibrations from the surface proteins of RGD-M13 phages, respectively [43–45]. Therefore, the RGD-GO-PLGA nanofiber mats were efficiently fabricated, and the RGD peptide and GO were successfully co-functionalized in the nanofibers.

Thermal behaviors of PLGA, GO-PLGA, RGD-PLGA and RGD-GO-PLGA nanofiber mats were analyzed by TGA to examine their thermal stabilities (Fig. 2b). TGA plots showed that the major weight losses occurred in the temperature ranges of 290–390°C, 320–405°C, 310–380°C and 310–370°C for PLGA, GO-PLGA, RGD-PLGA and RGD-GO-PLGA nanofiber mats, respectively, with negligible weight loss at temperature higher than 410°C. These major weight losses were appeared due to evaporation or thermal decomposition of the mats [24, 46]. This result implied that the thermal stability of RGD-GO-PLGA mats was not adversely affected by the co-functionalization of the RGD peptide and GO. Hence, it is proposed that the RGD-GO-PLGA mats can provide a thermally stable environment for supporting cell growth under the cell culture condition.

The degradation behavior is a significant factor to be considered for the design of the tissue engineering scaffold. Hence, we evaluated *in vitro* degradation behaviors of PLGA, GO-PLGA, RGD-PLGA, and RGD-GO-PLGA nanofiber mats by measuring the cumulative weight loss of nanofiber mats in DPBS at 37°C (Fig. 2c). All nanofiber mats begun to degrade after 1 week, and the mass loss gradually increased until 8 weeks. The weight loss of the RGD-PLGA nanofiber mats was the highest, followed by GO-PLGA, PLGA and RGD-GO-PLGA nanofiber mats. After 8 weeks, the cumulative weight losses were 28.33% for PLGA mats, 36.47% for GO-PLGA mats, 54.94% for RGD-PLGA mats and 20.59% for RGD-GO-PLGA mats. It has been reported that the improvement in surface hydrophilicity of the mats can promote water diffusion, which leads to the higher hydrolysis of PLGA fibers [47]. Therefore, RGD-PLGA nanofiber mats showed rapid degradation behavior because of the improved surface hydrophilicity. However, interestingly, RGD-GO-PLGA nanofiber mats exhibited slower degradation rate compared with the other mats. It could be due to the interfacial interaction between GO and RGD peptide or PLGA. Previous studies have documented that the oxygen-containing surface groups of GO, including carboxyl, hydroxyl, epoxy and carbonyl groups, can robustly interact with amine and hydroxyl groups of the mats [24, 25, 46, 48]. Therefore, the interfacial interaction between GO and RGD peptide or PLGA can decrease the degradation rate of the RGD-GO-PLGA nanofiber mats. Considering that the tissue engineering scaffolds should maintain adequate structural integrity to support cell growth during not only the *in vitro* cell culture but also subsequent *in vivo* implantation, although the optimal degradation rate has not been yet determined [49, 50]. Therefore, it is proposed that the RGD-GO-PLGA mats can be a suitable candidate for tissue engineering scaffold by providing a favorable environment for cell growth.

### Cellular behaviors of VSMCs on RGD-GO-PLGA nanofiber mats

**Figure 3:**
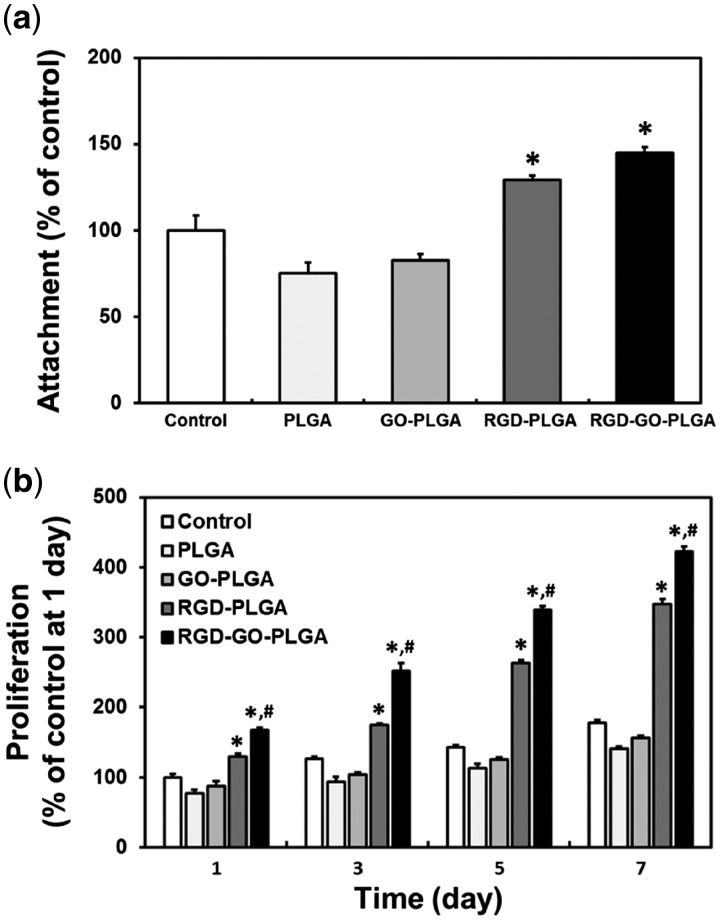
Cellular behaviors of VSMCs on RGD-GO-PLGA nanofiber mats. **(a)** Initial attachment and **(b)** proliferation of VSMCs on the control (TCPs), PLGA, GO-PLGA, RGD-PLGA and RGD-GO-PLGA nanofiber mats. An asterisk (*) denotes a significant difference compared with the control (*P* < 0.05), and a number sign (#) denotes a significant difference between the RGD-GO-PLGA nanofiber mats and the other groups (*P* < 0.05). The data are presented as the average ± SD of at least three independent experiments, each performed in duplicate on different cultures

The cellular behaviors of VSMCs on the RGD-GO-PLGA mats were examined by assessing the initial attachment and proliferation of VSMCs (Fig. 3). As shown in Figure 3a, the attachments of VSMCs on the RGD-PLGA and RGD-GO-PLGA nanofiber mats significantly (*P* < 0.05) increased than those on control (TCPs), PLGA and GO-PLGA nanofiber mats. It has been widely acknowledged that the RGD peptides play a fundamental role in cell attachment, and the RGD peptide-incorporated substrates can stimulate the cell attachment [51–55]. Therefore, the attachment of VSMCs was greatly increased on the RGD peptide-containing mats. Moreover, the proliferations of VSMCs on the RGD-PLGA and RGD-GO-PLGA nanofiber mats were significantly (*P* < 0.05) enhanced (Fig. 3b). These results are entirely consistent with previous studies showing that the RGD peptide-containing substrates can promote both initial attachment and proliferation through the robust activation of the integrin-mediated signaling pathway [54, 56]. In addition to this, the GO also participated in the enhanced proliferation of VSMCs. As shown in Figure 3b, the proliferation of VSMCs on the GO-PLGA nanofiber mats was slightly increased as compared with that on PLGA nanofiber mats. Interestingly, the proliferation of VSMCs on RGD-GO-PLGA nanofiber mats was further enhanced than that on only RGD peptide-functionalized PLGA nanofiber mats. This might be due to the ability of GO to promote cell growth. Previous studies have revealed that GO has a beneficial effect on the cellular growth by promoting initial attachment and proliferation [26, 57]. GO has many oxygen-containing functional groups on its basal plane and edges, including carboxyl, hydroxyl, epoxy and carbonyl groups, which enable to enhance the adsorption of RGD-GO-PLGA mats for the serum proteins in the culture media through electrostatic interactions [57, 58]. The adsorbed serum proteins can actively promote the cell growth. Hence, the proliferation of VSMCs was greatly promoted on the RGD-GO-PLGA mats as compared with the other mats because of the improved initial attachment and GO. These results indicated that the RGD peptide and GO can synergistically promote the cellular behaviors including initial attachment and proliferation. Therefore, it is proposed that the RGD-GO-PLGA mats can certainly provide a desirable microenvironment for cell growth by promoting both initial attachment and proliferation.

### Morphological analysis of VSMCs grown on RGD-GO-PLGA nanofiber mats

**Figure 4:**
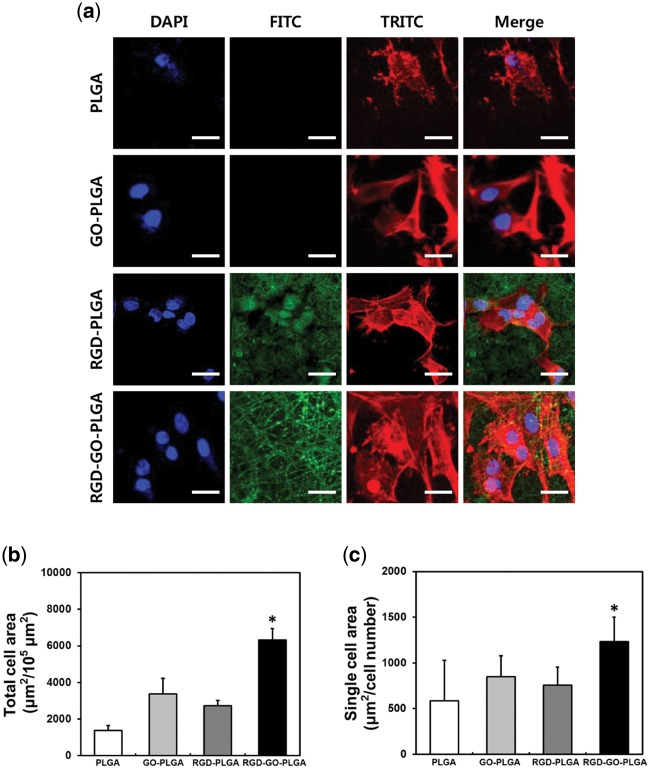
Morphological analysis of VSMC growth on RGD-GO-PLGA nanofiber mats. **(a)** Two-photon excitation fluorescence images of VSMCs on PLGA, GO-PLGA, RGD-PLGA and RGD-GO-PLGA nanofiber mats. F-actins of VSMC cytoskeletons were stained with TRITC-labeled phalloidin (red), cell nuclei were stained with DAPI (blue) and RGD-M13 phages in the nanofiber mats were immunofluorescence stained with the FITC-labeled α-M13 phage antibody (green). All images shown in this figure are representative of six independent experiments with similar results. The scale bars are 25 μm. **(b)** Quantification of the total cell area and **(c)** the single cell area of VSMC at 3 days after culture. The single cell area was calculated by dividing the total cell area by the total number of nuclei. Quantitative analysis was performed using ImageJ software. An asterisk (*) denotes a significant difference between the RGD-GO-PLGA nanofiber mats and the other groups (*P* < 0.05). The data are presented as the average ± SD of at least three independent experiments, each performed in duplicate on different cultures

To further examine the growth behaviors of VSMCs on the RGD-GO-PLGA nanofiber mats, the morphologies of VSMCs were analyzed by immunofluorescence staining (Fig. 4). The VSMCs were seeded on the PLGA, GO-PLGA, RGD-PLGA or RGD-GO-PLGA nanofiber mats and incubated for 3 days. As shown in Figure 4a, the morphology of VSMCs on PLGA nanofiber mat was apparently different from that on the other nanofiber mats. On the PLGA nanofiber mat, VSMCs showed shrunken morphology with poorly-organized F-actins and could not spread widely on the mat due to the hydrophobic property of PLGA [59]. On the contrary, VSMCs on the GO-PLGA, RGD-PLGA and RGD-GO-PLGA mats were observed to have spindle shape morphology with well-developed F-actins and were favorably grown on the mat. In particular, the number of VSMCs on the RGD-PLGA and RGD-GO-PLGA mats was apparently greater than PLGA and GO-PLGA mats, and the well-organized F-actins were clearly observed. These results are largely in accordance to the attachment and proliferation evaluation results, as presented in Figure 3. In addition, the RGD-PLGA and RGD-GO-PLGA nanofiber mats exhibited green fluorescence from functionalized RGD-M13 phages, indicating that the RGD peptides were evenly distributed in the throughout mats.

Furthermore, we quantified the cell area to reveal the detailed growth behavior of VSMCs on the nanofiber mats. As shown in Figure 4b, the total cell area of VSMCs cultured on RGD-GO-PLGA nanofiber mats significantly (*P* < 0.05) increased as compared with that on the other nanofiber mats, which could also be attributed to the synergistic effects of RGD peptide and GO. On the other hand, the single cell area of VSMCs on each nanofiber mat was shown in Figure 4c. The single cell area of VSCMs was also increased on GO-PLGA and RGD-PLGA nanofiber mats compared with that on PLGA nanofiber mats. Moreover, on RGD-GO-PLGA nanofiber mats, the single cell area was further significantly (*P* < 0.05) increased: 2.11-, 1.45- and 1.64-fold compared with that on PLGA, GO-PLGA and RGD-PLGA mats, respectively. These findings revealed that the RGD peptide and GO, co-functionalized within the mats, synergistically facilitated the cell adhesion on the mats leading to the enhanced cell spreading and growth [53, 54]. Hence, the RGD-GO-PLGA nanofiber mats can provide the most favorable microenvironment for cell growth. Taken together, it was demonstrated that the RGD-GO-PLGA nanofiber mats are capable of enhancing not only attachment and proliferation of VSMCs but also their growth behaviors. Hence, our results suggest that the RGD-GO-PLGA mats are demonstrated to sufficiently meet the requirements as tissue engineering scaffolds for directing cell growth.

## Conclusions

In the present study, we successfully fabricated RGD-GO-PLGA mats by electrospinning and investigated their physicochemical and thermal properties. It was shown that the RGD-GO-PLGA nanofiber mats have a three-dimensional network structure resembling the natural ECM, and the RGD peptide and GO were evenly co-functionalized in the mats. In addition, the physicochemical and thermal properties of RGD-GO-PLGA nanofiber mats are suitable for supporting cell growth. Moreover, the initial attachment and proliferation of VSMCs were markedly increased on the RGD-GO-PLGA nanofiber mats due to the synergistic effects of RGD peptide and GO. Furthermore, from morphological analysis, it was demonstrated that the growth behaviors of VSMCs were evidently improved on the RGD-GO-PLGA nanofiber mats, indicating that the RGD-GO-PLGA nanofiber mats are able to provide desired microenvironments for the growth of VSMCs. Collectively, it is proposed that the RGD-GO-PLGA nanofiber mats have a promising potential as biofunctional scaffolds for vascular tissue engineering.

## Funding

This study was supported by the Bio & Medical Technology Development Program of the National Research Foundation (NRF) funded by the Korean government (MEST) (No. 2015M3A9E2028643) and Basic Science Research Program through the NRF of Korea funded by the Ministry of Education (No. 2016R1D1A1B03931076).

*Conflict of interest statement*. None declared.
